# Cumulative Doses of T-Cell Depleting Antibody and Cancer Risk after Kidney Transplantation

**DOI:** 10.1371/journal.pone.0139479

**Published:** 2015-11-10

**Authors:** Jenny H. C. Chen, Germaine Wong, Jeremy R. Chapman, Wai H. Lim

**Affiliations:** 1 Department of Renal Medicine, Prince of Wales Hospital, Sydney, Australia; 2 Centre for Transplant and Renal Research, Westmead Hospital, Sydney, Australia; 3 Sydney School of Public Health, University of Sydney, Sydney, Australia; 4 Centre for Kidney Research, The Children’s Hospital at Westmead, Westmead, New South Wales, Australia; 5 Sir Charles Gairdner Hospital, Perth, Australia; Geisel School of Medicine at Dartmouth College, UNITED STATES

## Abstract

T-cell depleting antibody is associated with an increased risk of cancer after kidney transplantation, but a dose-dependent relationship has not been established. This study aimed to determine the association between cumulative doses of T-cell depleting antibody and the risk of cancer after kidney transplantation. Using data from the Australian and New Zealand Dialysis and Transplant Registry between 1997–2012, we assessed the risk of incident cancer and cumulative doses of T-cell depleting antibody using adjusted Cox regression models. Of the 503 kidney transplant recipients with 2835 person-years of follow-up, 276 (55%), 209 (41%) and 18 (4%) patients received T-cell depleting antibody for induction, rejection or induction and rejection respectively. The overall cancer incidence rate was 1,118 cancers per 100,000 patient-years, with 975, 1093 and 1377 cancers per 100,000 patient-years among those who had received 1–5 doses, 6–10 doses and >10 doses, respectively. There was no association between cumulative doses of T cell depleting antibody and risk of incident cancer (1–5: referent, 6–10: adjusted hazard ratio (HR) 1.19, 95%CI 0.48–2.95, >10: HR 1.42, 95%CI 0.50–4.02, p = 0.801). This lack of association is contradictory to our hypothesis and is likely attributed to the low event rates resulting in insufficient power to detect significant differences.

## Introduction

Monoclonal and polyclonal T cell depleting antibodies are utilized clinically as induction therapy to prevent acute rejection or as rescue therapy to treat steroid-resistant acute rejection in kidney transplantation [1]. However, T cell depleting antibodies are costly and may be associated with multiple complications, including infections and cancers [2, 3].

Trial-based evidence had shown an increased risk of malignancy by at least 2-fold with T-cell depleting antibodies compared with interleukin-2 receptor antibody (IL-2RAb) as induction therapy [1–3]. More recently, several large registry studies have shown a significant association between T cell depleting antibodies and increased risk of cancer, particularly post-transplant lymphoproliferative disease (PTLD) in kidney transplant recipients. Explorative analyses using the Collaborative Transplant Study (CTS) and the Australia and New Zealand Dialysis and Transplant (ANZDATA) registry reported the use of monoclonal and polyclonal T cell depleting antibodies as induction or as treatment for acute rejection is associated with over a 2 and 1.4-fold increased risk of incident cancer after transplantation respectively, suggesting T cell depletion may contribute to cancer development in kidney transplant recipients [3, 4].

Establishing a biological gradient between the exposure and outcome is an important criterion for causation in epidemiological research. Greater exposure may lead to greater incidence of the effect. To date, the association between dosing strategies and clinical complications such as infections and cancer after kidney transplantation remains unknown. In our study, we aimed to determine the association between the cumulative doses of T cell depleting antibodies used for induction or rejection and the risk of cancer after kidney transplantation.

## Materials and Methods

### Study population

Using the ANZDATA Registry, all primary live and deceased donor kidney transplant recipients in Australia and New Zealand between 1997 and 2012 were included. We excluded recipients receiving multiple organ grafts, recipients whose primary end-stage renal disease (ESRD) was caused by multiple myeloma or renal cell cancer, and those with a history of cancer prior to commencement of renal replacement therapy or while on maintenance dialysis prior to transplantation (except for non-melanocytic skin cancers). Recipients who received a kidney from donors with a history of cancer were excluded.

### T cell depleting antibody groups

T cell depleting antibody doses were stratified into tertiles, for all recipients who had received T cell depleting agents as induction therapy and/or treatment for acute rejection– 1–5 doses, 6–10 doses and >10 doses. Recipients who had received T cell depleting antibodies but had no records of the frequency of doses were excluded (n = 889). We included monoclonal and polyclonal T cell depleting antibodies in our analyses. Only the dose frequency of T cell depleting antibody is collected by the registry, However, the cumulative exposure of T cell depleting antibody (i.e. actual dose [expressed as total mg/dose or mg/kg/dose]) or the timing of the doses is not collected by the registry.

### Data collection

Recorded baseline data included donor age, type (live or deceased donor) and gender; recipients’ characteristics including age, gender, cause of ESRD (categorized as diabetic nephropathy, glomerulonephritis, cystic disease, vascular/hypertensive disease or others), pre-emptive transplants, peak panel reactive antibody (PRA), waiting time pre-transplant, diabetes, coronary artery disease (CAD) and smoking history (categorized as current smokers, former smokers or non-smokers); and transplant-related characteristics including human leukocyte antigen (HLA)-mismatches, ischaemic time, ABO-incompatible transplants, the use of other induction antibody therapy, number of rejection episodes and transplant era. Transplant era was divided into four groups for analysis (i.e. 1997–2000, 2001–04, 2005–08, 2009–12).

### Ascertainment of cancers

The ANZDATA registry records all incident cancers of kidney transplant recipients, except for squamous and basal cell cancers of the skin. Cancers reported to ANZDATA registry are coded for sites and cell type adapted from the International Classification of Disease for Oncology, first edition. It has been demonstrated that the cancer records within ANZDATA registry are robust and accurate, and previous analyses showed a high concordance rate when comparing the records of incident cancer diagnoses in patients on renal replacement therapy to those reported to the New South Wales Cancer Registry [5]. We included all cancers except non-melanocytic skin cancers, pre-malignant or in-situ lesions in our analyses.

### Statistical analyses

Comparisons of baseline characteristics between recipients who have received different doses of T cell depleting antibodies were examined by chi-square test and analysis of variance (ANOVA) for categorical and continuous variables respectively. For survival analyses, the follow up period was defined from the time of transplantation to the time of first cancer diagnoses after transplantation. Those who did not develop cancer were censored at the time of death or graft loss. The proportions free from incident cancers were calculated using the Kaplan-Meier Method. Results were expressed as hazard ratio (HR) or as odds ratio (OR) with 95% confidence intervals (CI). Covariates that were associated with cancer risk and had p-values of less than 0.2 in the unadjusted models were included in the adjusted models. All analyses were undertaken using SPSS V10 statistical software program (SPSS Inc., North Sydney, Australia) or SAS statistical software 9.4.

## Results

### Study population

Table 1 shows the baseline characteristics of the study population stratified by the tertiles of T cell depleting antibody dosing, with 182 (36.2%) receiving 1–5 doses, 234 (46.5%) receiving 6–10 doses and 87 (17.3%) receiving >10 doses. A total of 503 kidney transplant recipients between 1997 and 2012 were followed for a median of 4.4 years (IQR: 2.2 to 8.8 years) resulting in 2,835 person-years of follow-up. A total of 30 recipients (6.0%) developed cancers. The overall cancer incidence rate was 1,118 cancers per 100,000 patient-years, with 975 cancers per 100,000 patient-years among those who had received 1–5 doses of T cell depleting antibodies, 1,093 per 100,000 patient-years for those who had received 6–10 doses of T cell depleting antibodies and 1,377 per 100,000 patient-years for those who had received >10 doses of T cell depleting antibodies. Recipients who had received greater number of doses of T cell depleting antibodies were younger. Of those who had received T cell depleting antibodies for rejection, 30% had received monoclonal T cell depleting antibodies and 70% had received polyclonal T cell depleting antibodies (>95% received thymoglobulin). Of those who had received T cell depleting antibodies for induction, only 3% had received monoclonal T cell depleting antibodies. Recipients who had experienced vascular or glomerular rejection episodes were more likely to have received >5 doses of T cell depleting antibodies but the mean number of rejection episodes was similar between groups. The proportion of recipients who had experienced graft loss was significantly greater in those who have received >10 doses of T cell depleting antibodies compared to those who have received 1–5 and 6–10 doses (46%, 28% and 31% respectively, χ^2^ 9.03, p = 0.011). Of those who did not experience graft loss, cancer incidence in recipients who had received 1–5 doses, 6–10 doses and >10 doses was 3.8%, 3.7% and 4.3% respectively (χ^2^ 0.03, p = 0.986). Of those who had experienced graft loss, the incidence of cancer in recipients who had received 1–5 doses, 6–10 doses and >10 doses was 5.9%, 11.0% and 15.0% respectively (χ^2^ 2.06, p = 0.358). Only 4 recipients developed incident cancer after graft loss. Site-specific cancer frequencies stratified by tertiles of T cell depleting antibody doses are shown in Table 1.

**Table 1:** Baseline characteristics of kidney transplant recipients who have received T cell depleting antibodies (n = 503).

	1–5 doses (n = 182)	6–10 doses (n = 234)	>10 doses (n = 87)	P-value
Demographics				
Age	48.1±13.6	41.5±16.0	43.7±15.0	<0.001
Male	112 (61.5)	127 (54.3)	52 (59.8)	0.305
Pre-emptive	17 (9.4)	21 (9.0)	2 (2.3)	0.103
Diabetes	23 (12.6)	23 (9.8)	17 (19.5)	0.817
Coronary artery disease	25 (13.7)	23 (9.8)	7 (8.0)	0.286
Former/current smoker	83 (46.7)	89 (38.0)	36 (42.5)	0.319
Cause of ESRD				0.122
Glomerulonephritis	87 (47.8)	108 (46.2)	40 (46.0)	
Diabetes	18 (9.9)	17 (7.3)	14 (16.1)	
Cystic	29 (15.9)	27 (11.5)	11 (12.6)	
Waiting time (years)	3.8±3.2	3.4±3.4	4.1±3.3	0.205
Donor types				
Age	49.1±16.2	46.0±16.3	46.1±17.3	0.651
Male	72 (39.6)	96 (41.0)	40 (46.0)	0.584
Live-donor	57 (31.3)	77 (32.9)	19 (21.8)	0.151
Immunology/Transplant				
HLA-ABDR mismatches	3.4±1.7	3.6±1.7	3.5±1.6	0.646
ABO-incompatible	4 (2.2)	3 (1.3)	0 (0.0)	0.348
Peak PRA >50%	33 (18.2)	40 (17.1)	18 (20.7)	0.824
Ischaemic time	11.3±7.1	10.6±7.0	12.7±7.4	0.073
Induction				<0.001
IL-2RAb	53 (29.1)	127 (54.3)	29 (33.3)	
T cell depleting Ab	60 (32.9)	73 (31.2)	45 (51.7)	
Both	69 (37.9)	34 (14.5)	13 (14.9)	
Transplant era				<0.001
1997–2000	17 (9.3)	40 (17.1)	21 (24.1)	
2001–2004	21 (11.5)	60 (25.6)	29 (33.4)	
2005–2008	50 (27.5)	48 (20.5)	20 (23.0)	
2009–2012	94 (51.7)	86 (36.8)	17 (19.5)	
Outcomes				
Acute rejection	76 (41.8)	161 (68.8)	54 (62.1)	<0.001
Cellular	54 (29.7)	114 (48.7)	33 (37.9)	0.001
Glomerular	17 (9.3)	41 (17.5)	14 (16.1)	0.061
Vascular	34 (18.7)	79 (33.8)	27 (31.0)	0.003
Humoral^#^ (n = 315)	16 (11.1)	34 (25.4)	7 (18.9)	0.008
Number rejection	1.45±0.86	1.43±0.92	1.75±1.08	0.256
Graft failure	51 (28.0)	73 (31.2)	40 (46.0)	0.011
Incident cancer	8 (4.4)	14 (6.0)	8 (9.2)	0.298
Mean±SD years	4.61±3.74	5.62±4.12	6.91±3.98	<0.001
Median (IQR) years	3.55 (1.74, 7.02)	4.32 (2.05, 9.10)	6.36 (3.53, 10.04)	<0.001
Cancer types (n):				
Urogenital	2	4	0	
PTLD	1	2	1	
Lung	0	1	1	
Gastro-intestinal	1	1	0	
Prostate	1	0	1	
Breast	0	1	0	
Thyroid	0	1	1	

Data expressed as number (proportion) or as mean ± SD. ESRD–end-stage renal disease, HLA–human leukocyte antigen, PRA–panel reactive antibody, IL-2RAb–interleukin-2-receptor antibody, PTLD–post-transplant lympho-proliferative disease.

#Restricted to years 2005–2012.

### Use of T cell depleting antibodies for induction and/or rejection

Two hundred and seventy-six kidney transplant recipients (54.9%) received T cell depleting antibodies for induction, 209 (41.5%) received T cell depleting antibodies as treatment for rejection and 18 (3.6%) received T cell depleting antibodies both as induction and treatment for rejection. The mean and median doses of T cell depleting antibodies are shown in Table 2 with recipients who have received T cell depleting antibodies for induction and rejection being given up to twice the number of doses of T cell depleting antibodies compared to those who were given T cell depleting antibodies for induction or for rejection. The proportion of recipients who were given T cell depleting antibodies for induction and rejection and had developed cancer after transplantation was significantly higher compared to those who were given T cell depleting antibodies for induction or for rejection alone (22%, 7% and 3% respectively, χ^2^ 12.86, p = 0.002). Site-specific cancer frequencies including genito-urinary cancers and PTLD according to the use of T cell depleting antibodies are shown in Table 2.

**Table 2:** T cell depleting antibodies for induction and/or rejection.

	Induction (n = 276)	Rejection (n = 209)	Induction + Rejection (n = 18)
T cell depleting antibody doses			
Mean number of doses ±SD*	6.63±4.19	8.04±3.81	16.17±7.11
Median number of doses	6.00	8.00	14.50
(IQR)#	(3.00, 10.00)	(5.00, 10.00)	(11.00, 20.00)
Cancer incidence (n, %)^	20 (7.2)	6 (2.9)	4 (22.2)
Cancer type (n):			
Urogenital	4	2	1
PTLD	3	1	0
Lung	2	0	0
Gastro-intestinal	1	0	0
Prostate	1	1	0
Breast	1	0	0
Thyroid	1	1	0
Time to cancer (years)			
Mean±SD*	6.04±4.44	4.49±3.05	8.30±4.62
Median (IQR)#	4.55 (2.31, 9.60)	3.93 (1.83, 6.55)	8.70 (4.12, 12.62)

*1-way ANOVA p<0.001

#Kruskal-Wallis test p<0.001

^chi-square p<0.01

PTLD–post-transplant lympho-proliferative disease, SD–standard deviation, IQR–interquartile range

### Association between dose of T cell depleting antibodies and risk of incident cancer

**Fig 1:**
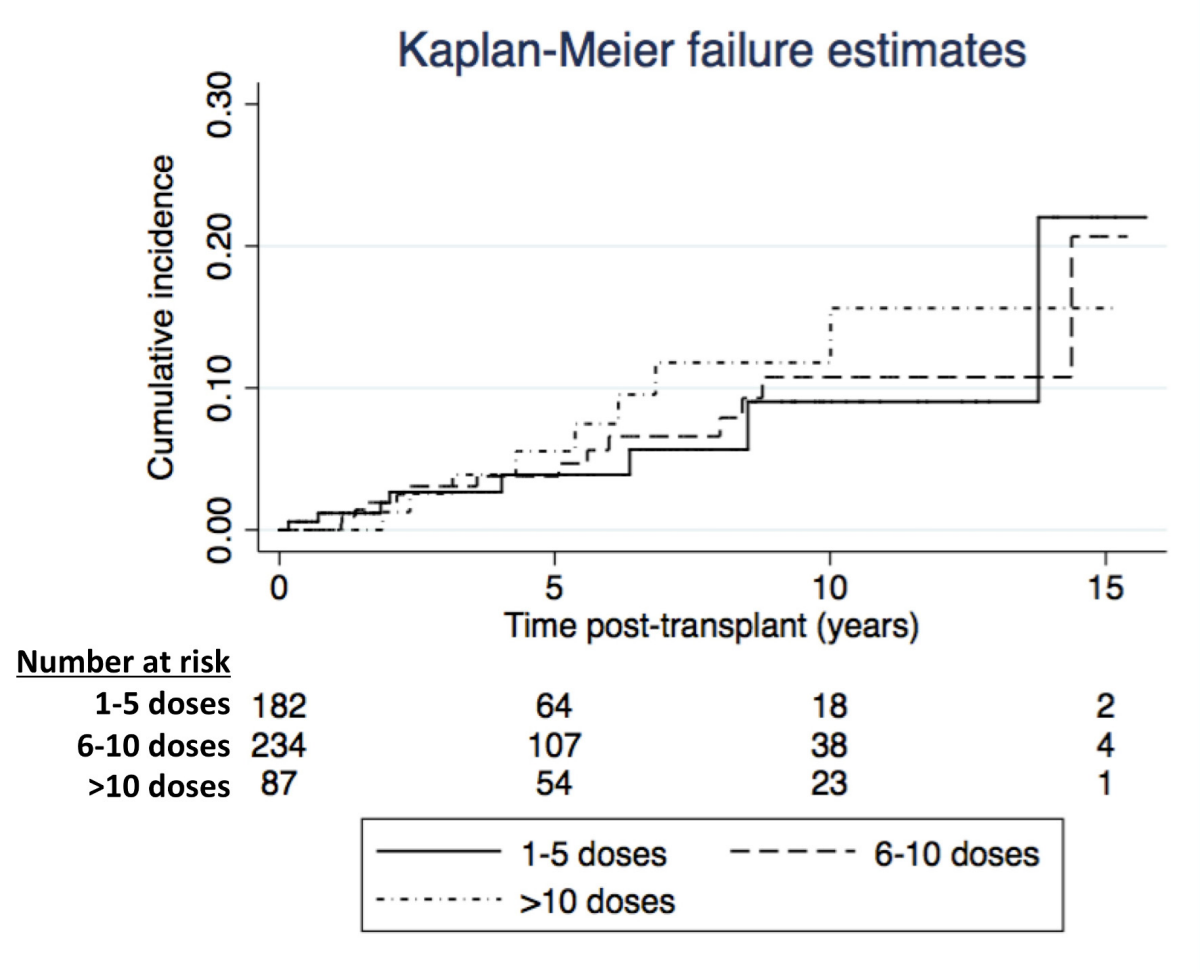
Adjusted cumulative cancer incidence stratified by tertiles of T-cell depleting antibody doses. (Log-rank p = 0.810).

Fig 1 shows the adjusted cumulative incidence of cancers stratified by tertiles of T cell depleting antibody dose (log-rank p-value 0.810). There was no association between T cell depleting antibody doses and risk of incident cancer (1–5 doses: referent, 6–10 dose: adjusted HR 1.19, 95% CI: 0.48, 2.95, >10 doses: adjusted HR 1.42, 95% CI: 0.50, 4.02, p-value for trend 0.801). In a separate model that included induction and/or rejection T cell depleting antibody use, there was no association with risk of incident cancer (induction: referent, rejection: adjusted HR 0.65, 95%CI: 0.23, 1.82, induction and rejection: adjusted HR 2.38, 95% CI: 0.77, 7.35, p-value for trend 0.163).

## Discussion

We have shown that kidney transplant recipients who were given a greater number of T cell depleting antibody doses, particularly those who were given T cell depleting antibodies for induction and rejection, had a higher incidence of cancer after transplantation. However, we were unable to show a significant association between incremental dose of T cell depleting antibodies and risk of incident cancer after kidney transplantation. The observed lack of dose-dependent response may reflect a small number of recipients included in our study and low event rates of cancer in kidney transplantation recipients.

Given the importance of T cells in the initiation of acute rejection, induction and maintenance immunosuppressive agents targeting T cell activation and function continues to remain the cornerstone of immunosuppressive regimens in kidney transplantation [6, 7]. It is well established that T cells are one of the major anti-tumour effector cells and therefore have a central role in anti-tumour surveillance [8]. Recent studies suggest that the balance between regulatory T cells, CD4+ T helper (Th)1 and CD8+ cytotoxic T cells may determine the development and prognosis of cancers in the general population and possibly in immunocompromised individuals [9, 10]. Dose-dependent lymphocytopaenia in the peripheral blood, spleen and lymph nodes has been shown following exposure to polyclonal T cell depleting antibodies in non-human primate model although this has not been conclusively shown in kidney transplant recipients [11, 12]. A large number of registry studies have shown that kidney transplant recipients who have received induction T cell depleting antibodies have a higher incidence of cancer after transplantation. Analysis of the CTS showed that the standardised incidence ratios (SIR) of lymphoma compared with a similar non-transplant population was almost 4-fold higher with the use of induction polyclonal T cell depleting antibodies (SIR 21.6, 95% CI 14.3–31.2; p<0.001) compared with IL-2RAb (SIR 7.8, 95% CI 4.4–12.9; p<0.001) or no induction (SIR 9.4, 95% CI 8.3–10.6 p<0.001) [3], a finding that has been corroborated with analysis of the Scientific Registry of Transplant Recipients (SRTR) and the United States Renal Data System databases [13, 14]. Similarly, a recent ANZDATA analysis of 7153 kidney transplant recipients showed that compared to recipients who did not experience acute rejection, those who were administered T cell depleting antibodies for treatment of acute rejection had a 1.4-fold greater risk of cancer, particularly genito-urinary tract cancers [4] suggesting that T cell depleting antibodies, whether used as induction or as treatment for rejection is associated with a heightened risk of incident cancer after transplantation. In a systematic review of randomized controlled trials comparing the risk and benefits of IL-2RAb and polyclonal T cell depleting antibodies as induction therapy, the use of polyclonal T cell depleting antibodies was associated with a 75% increase in the risk of cancer after transplantation [2], consistent with the findings of registry studies. However, none of these studies have reported on the dose of T cell depleting antibodies used and therefore the dose-dependent relationship between T cell depleting antibodies and risk of cancer after transplantation is unknown. In this study, we have shown a possible dose-dependent relationship between T cell depleting antibodies and risk of cancer after transplantation, with a higher incidence of cancer with incremental doses of T cell depleting antibodies, especially those who have received T cell depleting antibodies for induction and for rejection.

Monoclonal T cell depleting antibodies only deplete CD3+ T cells and is no longer commercially available, whereas the polyclonal T cell depleting antibodies not only target a variety of T and Natural Killer (NK) cell-derived antigens including CD2, CD3, CD4, CD8 and CD16, but they also target markers expressed by leukocytes, B cells and plasma cells [15–17]. Even though monoclonal and polyclonal T cell depleting antibodies may affect dissimilar populations of immune cells, clinical studies showed no difference in effectiveness between monoclonal and polyclonal T cell depleting antibodies in reversing rejection, preventing subsequent rejection or graft loss [1]. In addition, the relative risk of lymphoma after kidney transplantation was similar between induction with monoclonal or polyclonal T cell depleting antibodies [3]. In this study, we were unable to delineate the effects of individual T cell depleting agents because often, the different formulations of T cell depleting antibodies were not recorded in ANZDATA registry.

Our study has several strengths and limitations. To our knowledge, this is the first study that has explicitly explored the association between dose of T cell depleting antibodies and risk of overall cancers after primary kidney transplantation. The prospective nature and the completeness of the dataset in those with documented doses of T cell depleting antibodies suggest that selection and ascertainment biases in the exposure and study factors are minimized. However, the observed lack of association between doses and overall cancer risk is likely to reflect the small number of incident cancers in our cohort, and therefore there was insufficient power to detect any significant differences in overall cancer risk (i.e. potential for type II statistical error). Although multiple confounding factors were adjusted for, there may be unmeasured residual confounders such as the actual cumulative doses of T cell depleting antibodies, the intensity of concurrent maintenance immunosuppression and the extent of T cell depletion, all of which may have modified the association between dose and cancer risk. Selection bias may exist because there may be systematic differences in the treatment practices in induction and treatment of rejection between centres although decision to utilize T cell depleting antibodies for induction or rejection and the dosing regimens for T cell depleting antibodies are similar across transplanting centres within Australia and New Zealand. Given the small number of cancers in each group, evaluating the association between dose and site-specific cancers was not possible. We do acknowledge that the number of doses of T cell depleting antibody may not reflect the actual cumulative doses of this agent as the decision to use and prescribe/titrate the dose of T cell depleting antibody is dependent on multiple factors (e.g. physician preference of dose–multiple small doses vs single large dose, monitoring CD3 T cell subsets to determine dosing, presence of leucopenia, intensity of other immunosuppression, response to T cell depleting antibody), none of which are collected by ANZDATA registry and therefore it is plausible that the actual cumulative doses are similar between recipients who were prescribed different doses of T cell depleting antibody.

The incidence of cancer after transplantation was higher in kidney transplant recipients who have received a higher cumulative dose of T cell depleting antibodies, with almost 10% of those who had received over 10 doses of T cell depleting antibodies being diagnosed with cancer compared to 4% in those who have received less than or equal to 5 doses of T cell depleting antibodies. Although there was no significant association between incremental doses of T cell depleting antibodies and cancer risk, there was a trend towards a higher risk of cancer in those who have received a greater number of doses of T cell depleting antibodies, particularly those who have received antibodies for induction and treatment of rejection. However, given the shorter median follow-up period of recipients who have received higher doses of T cell depleting antibodies, longer-term follow-up of these recipients plus continuing data collection of all recipients who have received T cell depleting antibodies are essential to establish the association between dose of T cell depleting antibodies and cancer risk. Nevertheless, clinicians should be aware that T cell depleting antibodies are associated with a greater risk of cancer after transplantation and the continuing need to be vigilant and cognizant of the trade-off between incremental doses of T cell depleting antibodies and graft outcomes.
